# Reliability and validity of the international dementia alliance schedule for the assessment and staging of care in China

**DOI:** 10.1186/s12888-017-1544-3

**Published:** 2017-11-21

**Authors:** Xiao Wang, Zhenghai Sun, Lingchuan Xiong, Maya Semrau, Jianhua He, Yang Li, Jianzhong Zhu, Nan Zhang, Aimin Wang, Qinpu Jiang, Nan Mu, Yuping Zhao, Wei Chen, Donghui Wu, Zhanjie Zheng, Yongan Sun, Jing Zhang, Jun Xu, Xue Meng, Mei Zhao, Haifeng Zhang, Xiaozhen Lv, Norman Sartorius, Tao Li, Xin Yu, Huali Wang

**Affiliations:** 1 Dementia Care & Research Center, Peking University Institute of Mental Health, No.51 Huayuanbei Road, Haidian District, Beijing, 100191 China; 2 Key Laboratory for Mental Health, Ministry of Health (Peking University), Beijing, 100191 China; 3 Beijing Municipal Key Laboratory for Translational Research on Diagnosis and Treatment of Dementia, Beijing, 100191 China; 4 National Clinical Research Center for Mental Disorders (Peking University Sixth Hospital), Beijing, 100191 China; 5 School of Psychiatry, Qiqihar Medical University, Qiqihar, 161006 China; 6 Health Service and Population Research Department, Institute of Psychiatry, Psychology and Neuroscience, King’s College London, London, UK; 7 Department of Psychiatry of Beijing Anzhen Hospital Affiliated to Capital Medical University, Beijing, 100029 China; 8 Department of Neurology, The First Hospital of Shanxi Medical University, Taiyuan, 030001 China; 9 Wuxi Mental Health Center of Nanjing Medical University, Wuxi, 214151 China; 10 Department of Neurology, Tianjin Medical University General Hospital, Tianjin Neurological Institute, Tianjin, 300052 China; 11 Department of Neurology, The First Hospital of Changsha, Changsha, 410005 China; 12 Department of Geriatric Psychiatry, Hebei Mental Health Center, Baoding, 071000 China; 13 Department of Geriatric Psychiatry, Brain Hospital Affiliated to Guangzhou Medical University, Guangzhou, 510170 China; 14 Department of Geriatric Psychiatry, Shandong Mental Health Center, Jinan, 250014 China; 15 Department of Psychiatry, Sir Run Run Shaw Hospital, Zhejiang University School of Medicine, and the Collaborative Innovation Center for Brain Science, Hangzhou, 310016 China; 16 Department of Geriatric Psychiatry, Shenzhen Mental Health Center, Shenzhen, China; 17 Department of Geriatric Psychiatry, Qingdao Mental Health Center, Qingdao, 266034 China; 18 Department of Neurology, Peking University First Hospital, Beijing, 100034 China; 19 Department of Neurology, Beijing Tsinghua Changgung Hospital, Beijing, 102218 China; 20 Department of Neurology, Brain Centre, Northern Jiangsu Province Hospital, Affiliated to Yangzhou University, Yangzhou, 225001 China; 21 Association for the Improvement of Mental Health Programmes, Geneva, Switzerland

**Keywords:** IDEAL schedule, Validity, Reliability, Care, Dementia

## Abstract

**Background:**

Clinical and social services both are important for dementia care. The International Dementia Alliance (IDEAL) Schedule for the Assessment and Staging of Care was developed to guide clinical and social care for dementia. Our study aimed to assess the validity and reliability of the IDEAL schedule in China.

**Methods:**

Two hundred eighty-two dementia patients and their caregivers were recruited from 15 hospitals in China. Each patient-caregiver dyad was assessed with the IDEAL schedule by a rater and an observer simultaneously. The Clinical Dementia Rating (CDR), Mini-Mental Status Examination (MMSE), and Caregiver Burden Inventory (CBI) were assessed for criterion validity. IDEAL repeated assessment was conducted 7-10 days after the initial interview for 62 dyads.

**Results:**

Two hundred seventy-seven patient-caregiver dyads completed the IDEAL assessment. Inter-rater reliability for the total score of the IDEAL schedule was 0.93 (95%CI = 0.92-0.95). The inter-class coefficient for the total score of IDEAL was 0.95 for the interviewers and 0.93 for the silent raters. The IDEAL total score correlated with the global CDR score (ρ = 0.72, *p* < 0.001), the CDR-sum of box (CDR-SOB, ρ = 0.74, *p* < 0.001), the total score of MMSE (ρ = −0.65, *p* < 0.001) and CBI (ρ = 0.70, *p* < 0.001). All item scores of the IDEAL schedule were associated with the CDR-SOB (ρ = 0.17 ~ 0.79, all *p* < 0.05).

**Conclusion:**

The IDEAL schedule is a valid and reliable tool for the staging of care for dementia in the Chinese population.

**Electronic supplementary material:**

The online version of this article (10.1186/s12888-017-1544-3) contains supplementary material, which is available to authorized users.

## Background

Dementia is one of the biggest global public health problems for the elderly. Among the 46.8 million people worldwide now living with dementia, most live in low- or middle-income countries [1].In China, the disease burden and care demands of dementia have exponentially increased with the rapid growth of the population. However, no disease-modifying treatment exists [2]. Thus, dementia care is critical to maintaining a high quality of life during disease progression. Disease staging models that provide more precise information may be crucial to guide better person-centered care for persons with dementia in China.

Most of the current clinical staging scales, e.g., the Clinical Dementia Rating (CDR) scale [3], Global Deterioration Scale (GDS) [4], and Functional Assessment Staging (FAST) [5], focus on cognition or functional performance, without the inclusion of dementia care. Integrative assessment is one of the key components for psychosocial care of dementia [6]. A staging model including dementia care needs can provide more evidence for health care services. Therefore, it is imperative to include dementia care in the severity assessment framework.

The International Dementia Alliance (IDEAL) schedule was developed by the IDEAL study group based on the consensus on diagnosis and care of dementia patients. The IDEAL schedule stages dementia based on multiple perspectives, including social support and professional or informal care [7]. Use of the multidimensional schedule might improve the organization of regular or professional care for people with dementia. The IDEAL schedule is short and can be completed in 15 min. Health professionals, general practitioners, social workers and psychiatric nurses can use it. The IDEALschedule has been shown to have good content validity and high reliability in several countries [7–9]. However, the test-retest reliability has not been examined before. Besides, the Chinese version of IDEAL has not been validated. Testing the psychometric properties of Chinese IDEAL would provide more evidence for its application in dementia care in China.

Therefore, the present study first aimed to examine the psychometric property of the Chinese version of the IDEAL schedule, including internal consistency, test-retest reliability and inter-rater reliability. The secondary purpose was to evaluate the convergent validity of the schedule.

## Methods

### Research participants

From June to December 2015, 282 persons with dementia and their primary caregivers were recruited from memory clinics and neurology and psychiatry specialist clinics of 15 hospitals in 11 cities in China, including Beijing, Taiyuan, Guangzhou, Wuxi, Yangzhou, Tianjin, Changsha, Qingdao, Baoding, Shenzhen, and Hangzhou (see study raters and participating hospitals in Additional file 1). Persons with dementia and their informants were consecutively sampled.

Patients with dementia due to neurodegenerative diseases were consecutively recruited. A clinical diagnosis was made according to the criteria for dementia cited in the International Classification of Diseases, 10th Revision (ICD-10) [10]. To be included in the study, caregivers needed to visit the patients at least once a week. The exclusion criteria were as follows: The patient’s caregiver was not available, or the contact between caregiver and patient was insufficient (i.e., less than once a week); the patient was not able to speak Mandarin Chinese; the diagnosis remained unclear after diagnostic workup; the cognitive disorder was not due to neurodegenerative disease, e.g., brain tumour, delirium, etc.

The study was approved to be conducted in all participating hospitals by the institutional review board of Peking University Institute of Mental Health (Sixth Hospital). The ethics committee of Sir Run Run Shaw Hospital, Zhejiang University reviewed and additionally approved to conduct the study at Sir Run Run Shaw Hospital, Zhejiang University. Written informed consent was obtained from each patient-caregiver dyad. The patient and his/her legal guardian both provided written consent for the patient to participate in the study.

### Translation of IDEAL schedule

Two bilingual geriatric psychiatrists translated the IDEAL schedule from English to Mandarin Chinese. One professional translator reviewed the translation, made further changes and agreed on the semi-final Chinese version. Another independent professional translator translated the semi-final Chinese scale back into English, and the study team compared the translated and back-translated versions. After that, further changes were made to formulate the final Chinese version.

### Measures

To examine the convergent validity of individual items and the IDEAL schedule, we selected the following instruments as the reference:

#### Clinical dementia rating (CDR)

The CDR scale is a dementia staging instrument with good inter-rater reliability [11] and concurrent validity [12]. It consists of 6 domains: memory, orientation, judgment and problem solving, community affairs, home and hobbies, and personal care. Each domain scores from 0 (normal) through 3 (severe dementia), representing the different severity of dementia. The sum of box (CDR-SOB, range from 0 to 18) and an overall score of CDR were both used in the present study.

#### Mini-mental state examination (MMSE)

The MMSE [13] is widely used as a test for the general assessment of cognitive function. The test covers several cognitive domains, including orientation, immediate and short-term memory, concentration, naming, reading, comprehension, writing, and visual-motor abilities. The total score of MMSE ranges from 0 (severe impairment) to 30 (normal cognition).

#### Caregiver burden inventory (CBI)

The CBI is a 24-item inventory to evaluate caregiver burden. It consists of five factors: time-dependence burden, developmental burden, physical burden, social burden, and emotional burden [14].

### Rater training of IDEAL schedule

Twenty-six raters from15 sites received didactic training on how to administer the IDEAL schedule. The raters were asked to score for four cases examples after the training. During the training period, the intra-class correlation coefficients (ICC) of the total score of the IDEALschedule was 0.91.

### Procedures of assessment

For each dyad, two raters attended the IDEAL schedule interview: one of them conducted the interview (interviewer), and the other silently observed the interview (silent rater). The interviewer and the silent rater did not communicate and discuss the scores. Patients and caregivers were interviewed separately. The raters for MMSE, CBI and CDR were blind to the score of IDEAL. Sixty-two dyads repeated the IDEAL assessments7-10 days after the first interview.

### Statistical analyses

All analyses were performed with SPSS (version 16, SPSS Inc., Chicago, IL). Means and standard deviations were calculated for continuous data, and counts and prevalence rates for categorical data. Cronbach’s alpha coefficient and correlation coefficients between items were calculated to evaluate the internal consistency of the IDEAL schedule [15]. ICCs were used to assess the inter-rater reliability and the test-retest reliability [16]. For the concurrent validity, we used Spearman partial correlation analysis to examine the relationship between the individual items and the total score of the IDEAL schedule with the total score of the CDR-SOB, MMSE and CBI. The total score of IDEAL was also correlated with the global score of CDR.

## Results

A total of 277 patient-caregiver dyads (response rate of 98.23%) completed the assessment with the IDEAL schedule. As summarized in Table 1, 179 (64.6%) patients were female (mean age: 73.8 ± 8.7 years). Most patients were married (77.3%) and lived independently (96%). A total of 201 patients (71.5%) had received more than 9 years of schooling. Approximately 92.4% of caregivers were either spouses or children, and 148 (53.4%) caregivers were female. Majority of the patients were diagnosed with Alzheimer’s disease (*n* = 253, 91.3%). Other diagnoses included frontotemporal dementia (*n* = 9), Lewy body dementia (*n* = 6), Parkinson’s disease with dementia (*n* = 1), cerebral amyloid angiopathy (n = 2), and unspecified dementia (n = 6).

**Table 1:** Demographic and clinical characteristics of study participants

	Persons with dementia (*n* = 277)
Gender (male/female)	98/179
Age (years), mean (SD)	73.8 (8.7)
Degree of cognitive decline	
Very mild (CDR = 0.5)	51 (18.4%)
Mild (CDR = 1)	85 (30.7%)
Moderate (CDR = 2)	86 (31%)
Severe (CDR = 3)	55 (19.9%)
Marital status	
Married	214(77.3%)
Cohabiting	2 (0.7%)
Divorced	4 (1.4%)
Widowed/partner deceased	57 (20.6%)
Living arrangements	
Independent, alone, no day care	10 (3.6%)
Independent, alone, with day care	13 (4.7%)
Independent, with others, no day care	136 (49.1%)
Independent, with others, with day care	107 (38.6%)
Nursing home	7 (2.5%)
Other	4 (1.4%)
Level of education	
Fewer than 6 years of primary school	39 (14.1%)
6 years of primary school or special education school	37 (13.4%)
Secondary school education	80 (28.9%)
Vocational school (more than 9 years)	6 (2.2%)
Secondary professional education	32 (11.6%)
University / school completed at university entrance level	80 (28.9%)
Other	3 (1.1%)
Types of dementia	
Alzheimer’s disease	253 (91.3%)
Frontotemporal dementia	9 (3.2%)
Lewy body dementia	6 (2.2%)
Parkinson’s disease with dementia	1 (0.4%)
Cerebral amyloid angiopathy	2 (0.7%)
Unspecified dementia	6 (2.2%)

**Fig. 1:**
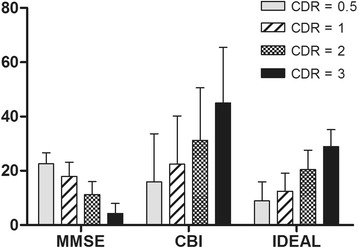
The scores of MMSE, CBI and IDEAL by the overall score of CDR. All *p* for group comparisons were <0.01. MMSE: mini-mental status examination; CBI: caregiver burden inventory; IDEAL: the International Dementia Alliance (IDEAL) schedule

As illustrated in Fig. 1, there were significant differences in the total scores of MMSE, CBI and IDEAL by the severity of dementia determined by the global score of CDR (CDR = 0.5, *n* = 51; CDR = 1, *n* = 85; CDR = 2, *n* = 86; CDR = 3, *n* = 55). The total score of the IDEAL schedule increased with dementia advancing.

### Internal consistency

The internal consistency, Cronbach’s alpha coefficient was 0.85. Table 2 shows correlation coefficients between individual items and the total IDEAL score. The correlation coefficients were satisfying for most individual items.

**Table 2:** Correlation of item scores for different dimensions in the IDEAL schedule

IDEAL Items	ADL	PH	CF	BPS	SS	T-NP	CD	H-PCR	H-PCN	T-DCN
ADL	–									
PH	0.21	–								
CF	0.77	0.21	–							
BPS	0.58	0.18	0.63	–						
SS	0.34	−0.07	0.26	0.30	–					
T-NP	0.63	0.10	0.55	0.52	0.28	–				
CD	0.52	0.13	0.48	0.57	0.23	0.53	–			
H-PCR	0.16	0.01	0.13	0.16	0	0.10	0.15	–		
H-PCN	0.40	0.08	0.41	0.45	0.18	0.35	0.53	0.42	–	
T-DCN	0.52	0.09	0.50	0.55	0.24	0.42	0.56	0.38	0.84	–
Total IDEAL	0.80	0.27	0.77	0.77	0.45	0.73	0.75	0.35	0.73	0.80

*ADL* activities of daily living, *PH* physical health, *CF* cognitive functioning, *BPS* Behavioural and psychological symptoms, *SS* social support, *T-NP* Time spent on care by non-professional care, *CD* Carer distress, *H-PCR* total number of hours of professional care received, *H-PCN* total number of hours of professional care needed, *T-DCN* type of dementia related care needed

### Inter-rater reliability

Table 3 shows the ICC coefficients (interviewer vs. silent rater) for the total score of the IDEAL schedule was 0.93 (95%CI = 0.92-0.95), indicating good inter-rater reliability. About 70% of the ICCs were higher than 0.8 (range 0.59-0.88), except for domain of physical health (0.72, 95% CI = 0.65-0.77), behavioural and psychological symptoms (0.79, 95% CI = 0.75-0.83), and social support (0.59, 95%CI = 0.51-0.66).

**Table 3:** Intra-class coefficients (ICC) of item scores and total score of the IDEAL schedule between interviewers and silent raters (inter-rater reliability)

IDEAL Items	ICCs (95%CI)
Activities of daily living	0.87 (0.84-0.90)
Physical health	0.72 (0.65-0.77)
Cognitive functioning	0.83 (0.79-0.86)
Behavioral and psychological symptoms	0.79 (0.75-0.83)
Social support	0.59 (0.51-0.66)
Nonprofessional care	
Time spent on care by non-professional carer	0.81 (0.77-0.85)
Carer distress	0.84(0.80-0.87)
Professional care	
Total number of hours of professional care received	0.87 (0.83-0.89)
Total number of hours of professional care needed	0.86 (0.83-0.89)
Type of dementia related care needed	0.88 (0.85-0.90)
Total IDEAL score	0.93 (0.92-0.95)

### Test-retest reliability

The ICC coefficient for the total score of IDEAL was 0.95 for the interviewers and 0.93 for the silent raters. For individual items, the test-retest reliability ranged between 0.72 and 0.96 for interviewers and between 0.67 and 0.94 for silent raters (see in Additional file 2: Table S1). The results indicated the rating of the IDEAL schedule was duplicable within 7-10 days.

### Convergent validity

Table 4 summarizes the correlation coefficients of the IDEAL schedule against validated instruments. The total score of IDEAL correlated with the CDR-global (ρ = 0.72, *p* < 0.001), the CDR-SOB (ρ = 0.74, *p* < 0.001), the total score of MMSE (ρ = −0.65, *p* < 0.001) and CBI (ρ = 0.70, *p* < 0.001). All item scores were associated with the CDR-SOB (ρ = 0.17 ~0.79, all *p* < 0.05). The total scores of MMSE and CBI were significantly correlated with most item scores except for physical health (Table 4). More correlation coefficients between the IDEAL individual items and CBI factors are shown in Additional file 3: Table S2.

**Table 4:** Correlation of total score of IDEAL, Chinese version, against validated instruments

IDEAL Items	CDR-SOB	MMSE	CBI
Activities of daily living	0.79*	−0.69*	0.54*
Physical health	0.17^#^	−0.08	0.10
Cognitive functioning	0.78*	−0.69*	0.48*
Behavioural and psychological symptoms	0.56*	−0.44*	0.51*
Social support	0.23*	−0.20^#^	0.30*
Time spent on care by non-professional carer	0.57*	−0.54*	0.52*
Carer distress	0.49*	−0.40*	0.66*
Total number of hours of professional care received	0.19^#^	−0.14^#^	0.15^#^
Total number of hours of professional care needed	0.45*	−0.42*	0.49*
Type of dementia related care needed	0.51*	−0.49*	0.53*
Total IDEAL score	0.74*	−0.65*	0.70*

* *p* < 0.001, ^#^ *p* < 0.05. *CDR-SOB* Clinical Dementia Rating - sum of box, *MMSE* Mini-Mental State Examination, *CBI* Caregiver Burden Inventory

## Discussion

The present study demonstrated adequate validity and reliability of the IDEAL schedule in staging dementia among Chinese people. Our study is the first one, to our knowledge, which evaluates the test-retest reliability of the IDEAL schedule.

The Cronbach’s alpha indicates that the IDEAL schedule has good structure validity. The finding is consistent with the previous study by Semrau et al. [7]. The IDEAL schedule intends to stage dementia based on clinical symptoms and care needs [7]. The assessment of social support and care needs is as important as other aspects to reflect an accurate picture of dementia care in practice.

Similar to studies on original version and among Irish and Spanish, the present study demonstrates moderate to excellent inter-rater reliability in Chinese [7–9]. The only concern lies in the relative low inter-rater reliability of assessment of social support level. The Spanish study ruled out the item of social support with factor analysis. Nevertheless, we recommend to include the level of social support in dementia staging system due to two main reasons. First, several studies have provided evidence that a low level of social support is an important risk factor for cognitive impairment [17–21]. Bennett and colleagues even argue that social networks modify the relation of some measures of Alzheimer’s disease pathology and level of cognitive function [22]. Second, social support is one of the key components of the psychosocial intervention of dementia care [6]. Social support is a multidimensional construct having perceived and objective elements [23]. Further studies need to provide supplemental definition and scoring anchors for a more reliable assessment of social support.

Compared with the Irish and Spanish studies, our finding adds test-retest reliability of the IDEAL schedule. Test-retest reliability is one of the important psychometric properties. Comparison of our results with previous studies is difficult because the test-retest reliability was not reported before [7–9]. The present study demonstrates adequate test-retest reliability for both interviewers and silent raters over an interval of 7-10 days. It supports the high stability of assessment with IDEAL over a short period.

Finally, the concurrent validity of the global IDEAL score when compared with the CDR-SOB and MMSE is similarly considered to be adequate. We observe a strong association between the total score of IDEAL and the CDR-SOB and MMSE. The finding is consistent with previous studies [7, 9]. Besides, the present study reveals a significant correlation between the total score of IDEAL, the carer distress item score and the total score CBI. In the Spanish study, Lopez-Anton et al. reported a correlation between care distress and the score of Zarit burden interview (ZBI) [9]. Our findings are comparable to the Spanish study and confirm that the IDEAL schedule could assess caregiver burden effectively.

An important limitation of this study is that participants were recruited at 15 sites across 11 cities in China, and researchers from different cities in different local cultures may have a different understanding of each domain of the IDEAL schedule, which may lead to interviewer bias. However, all raters from the participating sites had attended extensive training and supervision and reached high inter-rater reliability before starting the study. The qualification procedure is acceptable to minimize rater bias for multi-center clinical studies.

## Conclusion

This study indicates adequate validity and reliability of the Chinese version of the IDEAL schedule. The main psychometric properties of the IDEAL schedule confirm its application for clinical practice of dementia care in the Chinese population. To better assess the items of social support and care needs, further research is needed to provide precise definition and scoring anchors for individual items.

## Additional files

Additional file 1: Study groups, raters and participating hospitals (in alphabetic order by province or administrative city). (DOCX 15 kb)

Additional file 2: Table S1. Intra-class correlation coefficients (ICC) for item scores and the total score of IDEAL (test-retest reliability). (DOCX 25 kb)

Additional file 3: Table S2. Correlation of item scores of IDEAL, Chinese version, against factor scores of CBI. (DOCX 16 kb)
