# Supplementary material for: Reliability and validity of the international dementia alliance schedule for the assessment and staging of care in China
Source: BMC Psychiatry. 2017 Nov 21;17:371. doi: 10.1186/s12888-017-1544-3 (PMC5697421; doi:10.1186/s12888-017-1544-3)
Supplement: Supplementary file 1 — Study groups, raters and participating hospitals (in alphabetic order by province or administrative city). (DOCX 15 kb) [file 12888_2017_1544_MOESM1_ESM.docx]

Supplement 1. Study groups, raters and participating hospitals (in alphabetic order by province or administrative city):

Xiao Wang, Zhenghai Sun, Lingchuan Xiong, Meng Xue, Mei Zhao, Haifeng Zhang, Hongen Wei, Yanqin Wang (Peking University Sixth Hospital, Beijing, China); Jing Zhang, Ke Wang, Yuehong Xie (Beijing Tsinghua Changgung Hospital, Beijing, China); Hongxi Zhong, Huiting Lin, Peng Sun, Qianqian Han, Haixiao Guo, Heyu Lian, Huazhao Zhang, Hui Zhu, Enxian Li, Yan Zhang, Jie Zhang (Peking University First Hospital, Beijing, China); Jianhua He, Na Zhang, Congjia Li, Jing Zhang (Beijing Anzhen Hospital Affiliated to Capital Medical University, Beijing, China); Dongping Rao, Ruoyan Huang, Junchang Yu, Jie Dong (Brain Hospital Affiliated to Guangzhou Medical University, Guangzhou, Guangdong, China); Fei Feng, GuohuaXie, Yu Zang, Riyang Qiu, Zhiwei Wu (Shenzhen Mental Health Center, Shenzhen, Guangdong, China); Ying Zhou, Can Huang, Fei Xiao (The First Hospital of Changsha, Changsha, Hunan, China); Hongying Wang, Sujuan Wang, Zhihua Tong, Wei Li, Yanhua Wang (Hebei Mental Health Center, Baoding, Hebei, China); Jianzhong Zhu, Ping Ji, Bin Wu, Lifeng Tan (Wuxi Mental Health Center of Nanjing Medical University, Wuxi, Jiangsu, China); Lu Wang, Jiaxing Cheng, Fangyuan Chen, Jingjing Wang, Su Wu (Northern Jiangsu Province Hospital, Affiliated to Yangzhou University, Yangzhou, Jiangsu, China); Yang Li, Yan Lv, Jiyuan Li, Yarong Zhao, Hui Zhang (The First Hospital of Shanxi Medical University, Taiyuan, Shanxi, China); Jindong Wang, Bailing Wang, Lingli Kong (Qingdao Mental Health Center, Qingdao, Shandong, China); Yan Zhang, Yuping Zhao (Shandong Mental Health Center, Jinan, Shandong, China); Jinghao Shao, Mengya Xing, Changjuan Wei (Tianjin Medical University General Hospital, Tianjin Neurological Institute, Tianjin, China); Luoyi Xu, Lili We, LinlinTu, Yifei Li, Yingchun Zhang (Sir Run Run Shaw Hospital, Zhejiang University School of Medicine, and the Collaborative Innovation Center for Brain Science, Hangzhou, Zhejiang, China)
