# Supplementary material for: Reliability and validity of the international dementia alliance schedule for the assessment and staging of care in China
Source: BMC Psychiatry. 2017 Nov 21;17:371. doi: 10.1186/s12888-017-1544-3 (PMC5697421; doi:10.1186/s12888-017-1544-3)
Supplement: Supplementary file 2 — Intra-class correlation coefficients (ICC) for item scores and the total score of IDEAL (test-retest reliability). (DOCX 25 kb) [file 12888_2017_1544_MOESM2_ESM.docx]

Table S1. Intra-class correlation coefficients (ICCs) for item scores and the total score of IDEAL (test-retest reliability)

| **IDEAL items** | **Interviewers**  **(95%CI, n=62)** | **Silent raters**  **(95%CI, n=62)** |
| --- | --- | --- |
| Activities of daily living | 0.95 (0.92-0.97) | 0.84 (0.75-0.90) |
| Physical health | 0.88 (0.80-0.92) | 0.82 (0.72-0.89) |
| Cognitive functioning | 0.88 (0.80-0.92) | 0.83 (0.74-0.90) |
| Behavioural and psychological symptoms | 0.79 (0.75-0.83) | 0.87 (0.80-0.92) |
| Social support | 0.80 (0.70-0.88) | 0.67 (0.51-0.79) |
| Nonprofessional care |  |  |
| Time spent on care by non-professional carer | 0.96 (0.93-0.98) | 0.94 (0.90-0.96) |
| Carer distress | 0.72 (0.57-0.82) | 0.78 (0.65-0.86) |
| Professional care |  |  |
| Total number of hours of professional care received | 0.91 (0.86-0.95) | 0.86 (0.78-0.91) |
| Total number of hours of professional care needed | 0.96 (0.94-0.98) | 0.82 (0.72-0.89) |
| Type of dementia related care needed | 0.94 (0.90-0.96) | 0.80 (0.69-0.87) |
| Total score of IDEAL | 0.95 (0.92-0.97) | 0.93 (0.88-0.96) |

Notes: For ICCs, a two-way random absolute agreement single measures model was used. CI: confidence interval.
