# Supplementary material for: Reliability and validity of the international dementia alliance schedule for the assessment and staging of care in China
Source: BMC Psychiatry. 2017 Nov 21;17:371. doi: 10.1186/s12888-017-1544-3 (PMC5697421; doi:10.1186/s12888-017-1544-3)
Supplement: Supplementary file 3 — Correlation of item scores of IDEAL, Chinese version, against factor scores of CBI. (DOCX 16 kb) [file 12888_2017_1544_MOESM3_ESM.docx]

Table S2. Correlation of item scores of IDEAL, Chinese version, against factor scores of CBI

| **IDEAL Items** | CBI factors | | | | |
| --- | --- | --- | --- | --- | --- |
|  | Time-dependence burden | Developmental burden | Physical burden | Social burden | Emotional burden |
| Activities of daily living | 0.68^*^ | 0.49^*^ | 0.38^*^ | 0.31^*^ | 0.33^*^ |
| Physical health | 0.17^#^ | 0.11 | 0.05 | 0.05 | -- |
| Cognitive functioning | 0.58^*^ | 0.44^*^ | 0.33^*^ | 0.28^*^ | 0.33^*^ |
| Behavioural and psychological symptoms | 0.50^*^ | 0.49^*^ | 0.39^*^ | 0.39^*^ | 0.32^*^ |
| Social support | 0.31^*^ | 0.28^*^ | 0.28^*^ | 0.21^#^ | 0.17^#^ |
| Time spent on care by non-professional carer | 0.62^*^ | 0.48^*^ | 0.44^*^ | 0.27^*^ | 0.25^*^ |
| Carer distress | 0.54^*^ | 0.65^*^ | 0.57^*^ | 0.50^*^ | 0.49^*^ |
| Total number of hours of professional care received | 0.14^#^ | 0.11 | 0.11 | 0.14^#^ | 0.10 |
| Total number of hours of professional care needed | 0.44^*^ | 0.43^*^ | 0.40^*^ | 0.44^*^ | 0.36^*^ |
| Type of dementia related care needed | 0.50^*^ | 0.50^*^ | 0.42^*^ | 0.46^*^ | 0.36^*^ |

* *p* < 0.05. CBI: Caregiver Burden Inventory.
